# *In silico* serotyping of *E. coli* from short read data identifies limited novel O-loci but extensive diversity of O:H serotype combinations within and between pathogenic lineages

**DOI:** 10.1099/mgen.0.000064

**Published:** 2016-07-11

**Authors:** Danielle J. Ingle, Mary Valcanis, Alex Kuzevski, Marija Tauschek, Michael Inouye, Tim Stinear, Myron M. Levine, Roy M. Robins-Browne, Kathryn E. Holt

**Affiliations:** ^1^​ Department of Microbiology and Immunology, Peter Doherty Institute for Infection and Immunity, University of Melbourne, Parkville, Victoria 3010, Australia; ^2^​ Centre for Systems Genomics, University of Melbourne, Parkville, Victoria 3010, Australia; ^3^​ Department of Biochemistry and Molecular Biology, Bio21 Molecular Science and Biotechnology Institute, University of Melbourne, Parkville, Victoria 3010, Australia; ^4^​ Microbiological Diagnostic Unit Public Health Laboratory, Peter Doherty Institute for Infection and Immunity, University of Melbourne, Victoria 3010, Australia; ^5^​ School of BioSciences, University of Melbourne, Victoria 3010, Australia; ^6^​ Department of Medicine, University of Maryland School of Medicine, Baltimore, MD 21201, USA; ^7^​ Murdoch Childrens Research Institute, Royal Children's Hospital, Victoria 3010, Australia

**Keywords:** *E. coli*, serotype, genotype, phenotype, diversity

## Abstract

The lipopolysaccharide (O) and flagellar (H) surface antigens of *Escherichia coli* are targets for serotyping that have traditionally been used to identify pathogenic lineages. These surface antigens are important for the survival of *E. coli* within mammalian hosts. However, traditional serotyping has several limitations, and public health reference laboratories are increasingly moving towards whole genome sequencing (WGS) to characterize bacterial isolates. Here we present a method to rapidly and accurately serotype *E. coli* isolates from raw, short read WGS data. Our approach bypasses the need for *de novo* genome assembly by directly screening WGS reads against a curated database of alleles linked to known and novel *E. coli* O-groups and H-types (the EcOH database) using the software package srst2. We validated the approach by comparing *in silico* results for 197 enteropathogenic *E. coli* isolates with those obtained by serological phenotyping in an independent laboratory. We then demonstrated the utility of our method to characterize isolates in public health and clinical settings, and to explore the genetic diversity of >1500 *E. coli* genomes from multiple sources. Importantly, we showed that transfer of O- and H-antigen loci between *E. coli* chromosomal backbones is common, with little evidence of constraints by host or pathotype, suggesting that *E. coli ‘*strain space’ may be virtually unlimited, even within specific pathotypes. Our findings show that serotyping is most useful when used in combination with strain genotyping to characterize microevolution events within an inferred population structure.

## Data Summary

1. The EcOH database of O- and H-antigen sequences is distributed with srst2 (https://github.com/katholt/srst2). Sequences used in the EcOH database are given in Table S1, available in Figshare https://dx.doi.org/10.4225/49/571996C105E03
2. All raw sequence data (reads and/or assembled genomes) for the *E. coli* genomes analysed in this publication are publicly available under the project numbers given in Table 1. These are: EPEC http://www.ebi.ac.uk/ena/data/view/ERP001141; ETEC in several projects http://www.ebi.ac.uk/ena/data/view/ ERP000733, http://www.ebi.ac.uk/ena/data/view/ERP000270, http://www.ebi.ac.uk/ena/data/view/ERP001592, http://www.ebi.ac.uk/ena/data/view/ERP002086, http://www.ebi.ac.uk/ena/data/view/ERP000349, http://www.ebi.ac.uk/ena/data/view/ERP001074; UPEC http://www.ebi.ac.uk/ena/data/view/ERP001354, http://sra.dnanexus.com/studies/SRP027327; and GenomeTrakr http://www.ncbi.nlm.nih.gov/bioproject/183844.
3. Interactive versions of all phylogenetic trees shown are available in MicroReact: UPEC ST131, http://microreact.org/project/Ny5Gg4Wg-; GenomeTrakr, http://microreact.org/project/VygdKU_0. Tree files (Newick format) and metadata (CSV format) are also available for download via these links.
4. All other supplementary information is available in Figshare (https://dx.doi.org/10.4225/49/571996C105E03). These include the NCBI results from Table S2 (PDF format), sequences and annotations for novel O loci identified (GenBank format), validation of phenotype from genotype on the 197 EPEC isolates in Tables S3–5 (PDF format), diversity results on 1547 *E. coli* are summarized in Table S6 (Excel format) and Supplementary Figures from this study as a single PDF.

## Impact Statement

We present an analysis of the diversity of O- and H-types within *E. coli*. To do this we developed and validated a method to determine these O- and H-types rapidly and accurately from short read whole genome sequence (WGS) data using the program srst2. We show that our method has advantages (i) over serological phenotyping, which leaves many isolates non-typeable, and (ii) over assembly-based approaches to *in silico* serotyping using WGS data. We show how our method can readily be combined with rapid inference of other important attributes of *E. coli*, including MLST and antibiotic resistance profile. Furthermore, we identified 38 novel putative O-antigens resulting in the expansion of the EcOH database. Our method will have important implications for characterizing *E. coli* isolates of public health relevance on a large scale. We found that while there are a limited number of O- and H-types, the potential combination of O-type, H-type and chromosomal lineages is vast. Applying our method to the determination of MLST and serotypes together from WGS data, we found that *E. coli* serotypes can change within lineages, resulting in different sub-lineages that facilitate tracking of pathogenic *E. coli* over time, including during outbreaks.

## Introduction

*Escherichia coli* is a Gram-negative bacillus that is a gut commensal, as well as a leading cause of diarrhoea, foodborne outbreaks globally and various extra-intestinal infections. Differentiation of *E. coli* isolates has traditionally been performed by serological typing (serotyping) of the highly polymorphic somatic- (O) and flagellar- (H) antigens to identify pathogenic lineages of *E. coli* (pathotypes) (DebRoy *et al.*, 2011; Robins-Browne, 1987; Wang *et al.*, 2003). The O-antigen is an integral part of the lipopolysaccharide in the outer membrane of Gram-negative bacteria, whilst the H-antigen (flagellin) projects beyond the cell wall and provides cell motility, which allows the bacteria to reach their preferred ecological niche (Li *et al.*, 2010; Wang *et al.*, 2003). Currently there are 182 *E. coli* O-groups and 53 H-types recognized by traditional serotyping (Croxen *et al.*, 2013; Iguchi *et al.*, 2014; Joensen *et al.*, 2015). Several serotypes are considered to be markers of pathogenic *E. coli* and are routinely screened for in public health and food industry settings. The value of serotyping is illustrated by the discovery of enteropathogenic *E. coli* (EPEC), which was first described as a group of antigenically related *E. coli* isolates associated with outbreaks of infantile diarrhoea (Robins-Browne, 1987). One of the best characterized *E. coli* serotypes is O157:H7, which is associated with enterohaemorrhagic *E. coli* (EHEC) strains that have caused multiple foodborne outbreaks of diarrhoea, haemorrhagic colitis and haemolytic uraemic syndrome since 1982 (Dallman *et al.*, 2015; Michino *et al.*, 1999; Riley *et al.*, 1983). Serotyping is still relied upon to characterize such outbreaks. For example, during an investigation of a large foodborne outbreak of Shiga toxin-producing *E. coli* (STEC) in Germany in 2011 (Rasko *et al.*, 2011), its serotype, O104:H4, was used as the marker of the outbreak strain (Frank *et al.*, 2011a, b).

Biosynthesis of the O-antigen in *E. coli* is encoded in gene clusters that are typically located between the chromosomal housekeeping genes *galF* and *gnd*/*ugd* (Iguchi *et al.*, 2014; Liu *et al.*, 1996). The genes required to synthesie this antigen fall into three classes: (i) sugar synthesis genes, (ii) glycosyl transferase genes and (iii) O-antigen processing genes (Samuel & Reeves, 2003). Two distinct biosynthetic O-antigen pathways are known: (i) the Wzx/Wzy-dependent pathway, encoded by the *wzx* (O-antigen flippase) and *wzy* (O-antigen polymerase) genes, and (ii) the ABC transporter pathway, encoded by *wzm* and *wzt* (Feng *et al.*, 2004; Samuel & Reeves, 2003). In general, variation in these gene sequences correlates with structural variation in the carbohydrate residues that make up each O-antigen (DebRoy *et al.*, 2011; Wang *et al.*, 2003). Because of this, the sequences of these genes can be used for O-antigen genotyping (Joensen *et al.*, 2015; Mentzer *et al.*, 2014). Nevertheless, genotype–phenotype relationships for some O-groups are unexpectedly complex. For example, two distinct gene clusters are associated with the same O45 antigen (Plainvert *et al.*, 2007), whereas some distinct O-antigens are encoded by near-identical gene clusters (Iguchi *et al.*, 2014).

H-antigen specificity is determined by flagellin, which is the protein subunit of flagella. This protein is encoded by *fliC* in 43 of the 53 serologically defined H-types (Wang *et al.*, 2003). PCR detection of *fliC* alleles has been used for molecular H-typing for some time (Wang *et al.*, 2003). However, some *E. coli* isolates have an alternative flagellar phase, due to the presence of an additional flagellin gene (*flnaA*, *fllA*, *fmlA* or *flkA*), similar to those found in *Salmonella* species (Feng *et al.*, 2008; Ratiner, 1998; Ratiner *et al.*, 2010; Tominaga, 2004; Tominaga & Kutsukake, 2007).

As the number of possible combinations of known *E. coli* O- and H-antigens is over 10 000, serotype is often assumed to be a marker for specific types or lineages of *E. coli*. However, the O-antigen region is subject to strong selection pressure from the immune systems of mammalian hosts and also from predation by bacteriophages, resulting in large numbers of recombination events around the O-antigen locus. Thus, it is possible for two isolates from unrelated *E. coli* lineages to have converged upon the same serotype, or for two closely related isolates to have different serotypes.

Determination of serotype involves performing a series of agglutination reactions with panels of antisera, and is expensive in terms of both labour and reagent costs (Achtman *et al.*, 2012; Fratamico *et al.*, 2009). In addition, the interpretation of these assays is subjective and relies on antisera that vary in titre and specificity. Furthermore, a significant proportion of *E. coli* isolates (approximately one-quarter) are serologically ‘untypeable’ due mainly to autoagglutination or lack of reaction with available antisera (DebRoy *et al.*, 2011). For these reasons there has been a shift away from serological phenotyping of *E. coli*, towards inference of O- and H-genotypes using molecular methods (Jenkins, 2015). To date, the molecular methods have predominately been PCR-based approaches targeting the *wzx*/*wzy* or *wzm*/*wzt* variants.

As the cost of high-throughput short read DNA sequencing declines, public health laboratories are increasingly moving away from phenotyping and towards whole genome sequence (WGS)-based typing of bacteria, including *E. coli* (Joensen *et al.*, 2015; Kwong *et al.*, 2015). Given the strong genetic basis for O- and H-antigenic variation in *E. coli*, the availability of genome data provides an opportunity to infer serotypes at little or no additional cost. Joensen *et al.* (2015) reported SeroFinder, a web-based tool for the inference of *E. coli* serotypes via blast analysis of *de novo* assembled WGS data. While that study demonstrated convincingly the principle that inference of serotype from WGS data is feasible, SeroFinder does not provide the rapid and robust high-throughput screening required by public health laboratories. Firstly, the reliance on *de novo* assembly compromises robustness, as the generation of high-quality assemblies from short read data is difficult to standardize, and we have previously shown that the detection of gene content from such data is less sensitive than direct read analysis with srst2 (Inouye *et al.*, 2014). Secondly, SeroFinder is a web-based tool that requires data to be uploaded to a server in Denmark. While this facilitates small-scale analyses by bypassing the need for local software installation, it is unsuitable for high-throughput use and cannot be integrated into users’ existing bioinformatics pipelines.

Here we present a method to rapidly and accurately serotype *E. coli* isolates directly from WGS reads, using srst2 to screen reads directly against a curated database of alleles linked to known *E. coli* O-groups and H-types (the EcOH database). We validated our approach through comparison with both independent serological phenotyping and genotyping by blast analysis of *de novo* assembled WGS data, and then demonstrated the utility of our approach for rapid typing of epidemic clones and foodborne outbreaks. Applying our approach to explore serotype diversity in a wide range of *E. coli* isolates revealed 38 novel O-antigen loci, expanding previously published typing sets by 20 %.

## Methods

### Curation of the EcOH database.

The EcOH database of O- and H-type encoding sequences was initially constructed in 2014 from publicly available sequences identified in GenBank by reviewing the literature on the PCR detection of *E. coli* O- and H-types (DebRoy *et al.*, 2011; Ratiner *et al.*, 2010; Wang *et al.*, 2003). This was updated by a further review in May 2015 (Iguchi *et al.*, 2014; Joensen *et al.*, 2015). Novel O-loci identified in the present study were also included. The resulting EcOH database includes sequences of alleles of *wzm* and *wzt*, or *wzx* and *wzy,* covering 180 established O-groups (of a possible 182). The two exceptions were O57 and O14, as isolates with these O-groups lack any of these genes and harbour only small O-antigen gene clusters, with no known polymerase or flippase genes; and only the housekeeping genes *galF*, *gnd* and *hisI* together with *ugd* and *wzz*, which are not sufficient to delineate these O-groups. The EcOH database also includes sequences for all 53 known H-types, allowing for the detection of both *fliC* and non-*fliC* flagellin (*flnA*, *fmlA*, *flkA* and *fllA*) genes, and for the identification of isolates that may be able to undergo flagellum phase variation (Tominaga, 2004; Tominaga & Kutsukake, 2007). For the O-groups, the database specifies the 16 O-group clusters containing 37 O-groups as identified by Iguchi *et al.*(2014) where there was >95 % sequence identity. The number of total O-group/clusters is 161. Details of all sequences in the EcOH database are provided in Table S1 (available in the online Supplementary Material). The EcOH database is available at https://github.com/katholt/srst2

### Publicly available sequence data used in this study.

Details of the short read Illumina data used in this study are provided in Table 1. A total of 41 complete *E. coli* genomes were downloaded from PATRIC (Wattam *et al.*, 2013), with the accession numbers listed in Table S2. Serologically determined O-groups were identified in the GenBank entries or associated literature for 40 of these genomes (and H-types for 20) (Table S2). The Achtman MLST scheme for *E. coli* (Wirth *et al.*, 2006), now hosted at Warwick University (http://mlst.warwick.ac.uk/mlst/dbs/Ecoli), was downloaded using the *getmlst.py* script included in the srst2 package (https://github.com/katholt/srst2). An srst2-formatted version of the ARG-ANNOT antimicrobial resistance gene database (Gupta *et al.*, 2013) was downloaded from https://github.com/katholt/srst2

**Table 1.** Datasets used to assess accuracy and utility of the EcOH database

Isolates	Citation	ENA or NCBI project number	No. of genomes	Population	Sequencing centre
EPEC (atypical EPEC)	Ingle *et al.* (2016)	ERP001141 (ENA)	197 (185)	Diverse	Sanger, UK
UPEC	Petty *et al.* (2014), Price *et al.* (2013)	ERP001354 (ENA) SRP027327 (NCBI)	169	Clonal, ST131	UQ, Australia Arizona, USA
GenomeTrakr		PRJNA183844 (NCBI)	300	Diverse	Food and Drug Administration, USA
GenomeTrakr		PRJNA183844 (NCBI)	1000	Diverse	Food and Drug Administration, USA
ETEC	Mentzer *et al.* (2014)	Various, see Mentzer *et al.* (2014)	362	Diverse	Sanger, UK

### Assembly and blast analysis.

Illumina reads of 100 bp paired end were generated previously for 197 EPEC isolates (Ingle *et al.*, 2016) and assembled using Velvet and Velvet Optimiser (Zerbino & Birney, 2008). Reads and assemblies are available in the European Nucleotide Archive (ENA) under ERP001141 (Ingle *et al.*, 2016). Here, we generated alternative assemblies using SPAdes (v3.0) (Bankevich *et al.*, 2012) with error correction and kmer lengths of 21, 33, 55, 77 and 89. The resulting contigs were extended with the scaffolder sspace (Boetzer *et al.*, 2011); gaps within the scaffolds were closed using GapFiller (Boetzer & Pirovano, 2012) and then further extended with AlignGraph (Bao *et al.*, 2014).

Both sets of assemblies were screened against the EcOH database using blast+ (*blastn*). A genotype call was made where a hit was identified with ≥90 % coverage of a query sequence at ≥90 % nucleotide identity. Note that as the SPAdes assemblies yielded fewer genomes with blast+ hits than O-antigen loci, these assemblies were discarded and all results were reported as comparisons of srst2 data to assembly-based analysis using the Velvet Optimiser assemblies. This made the comparison as favourable as possible towards the competing method of assembly-based analysis.

Publicly available short reads for 64 enterotoxigenic *E. coli*(ETEC) (Mentzer *et al.*, 2014) and 162 GenomeTrakr isolates that were O-non-typeable from the EcOH database were assembled with SPAdes (v3.6) (Bankevich *et al.*, 2012), ordered with abacas (Assefa *et al.*, 2009) against the reference *E. coli* 12009 O103:H2 genome, and annotated with Prokka (v1.11) (Seemann, 2014).

### srst2 analysis.

The program srst2 was run with default parameter settings, such that a genotype call reflected detection of reads covering ≥90 % of the length of a query locus at ≥90 % nucleotide identity. Where multiple alleles of the same locus appeared in the database, srst2 reported the best-scoring allele as the genotype call (Inouye *et al.*, 2014). A confident genotype call was defined as one exceeding the minimum depth cut-offs (Inouye *et al.*, 2014). Here we used the srst2 default values of ≥5-fold mean read depth across the query locus to define a confident call.

### Phenotypic characteriation of 197 EPEC isolates.

A total of 197 EPEC isolates were available for analysis, including 185 atypical EPEC (aEPEC) and 11 typical EPEC from the Global Enteric Multi-Center Study (GEMS) (Ingle *et al.*, 2016), and one aEPEC isolate from Melbourne, Australia. Isolates were subcultured on Luria–Bertani agar and incubated overnight at 37 °C before being submitted to the National *E. coli* Reference Laboratory at the Microbiological Diagnostic Unit Public Health Laboratory (MDU PHL) in Melbourne, Australia, for serotyping. O-and H- serotyping utilized the standard tube agglutination tests, adapted for U-bottomed microtitre trays (Chandler & Bettelheim, 1974). As controls, all O- and H-antigen agglutination reactions were titrated in parallel with the corresponding internationally recognized control strain. Results were accepted when the antibody titre for the control antigen was within one dilution of the expected titre results based on antisera quality control records, when the titre of the test strain was within one dilution of the control antigen titre and when the negative control did not produce any agglutination with the test or control. Agglutination observed with the negative control was considered ‘O-rough’ and thus untypeable by this method. No agglutination reaction with any antisera in the panel was deemed ‘O-non-typeable’.

### Characterization of potential novel O-antigens in GEMS isolates.

Where an O-group was determined via serological testing of an EPEC isolate, but no known *wzx*/*wzy* or *wzm*/*wzt* genes were detected in the corresponding isolate’s genome, the *de novo* genome assembly was interrogated to identify potential novel O-antigen loci. For each such EPEC isolate the assembled contig containing the genes *galF* and *gnd*, which typically flank the O-antigen locus, were identified using blast and extracted using emboss (Rice *et al.*, 2000). The intervening sequences were annotated with Prokka (v1.11) (Seemann, 2014), using translated protein sequences from the EcOH database as the preliminary annotation source. We then used act (Carver *et al.*, 2008) to visually compare the annotated sequences with full-length reference sequences for the corresponding O-group that had been identified by serology. Putative *wzx* and *wzy* alleles for these O-groups were identified based on (i) the annotation, (ii) sequence homology with the reference O-group sequences and (iii) the presence of transmembrane domains identified using tmhmm (Krogh *et al.*, 2001). These putative *wzx* and *wzy* gene sequences from six novel O-antigen loci were added to the EcOH database with the suffix ‘var1', 'var2’, etc., to differentiate them from the prototypical alleles. For example, the novel *wzx* gene detected in isolates that were serologically phenotyped as O116 are labelled ‘*wzx*-O116var1’, whereas the prototypical O116 *wzx* gene is labelled *wzx*-O116 (Table S1).

### Analysis of ST131 uropathogenic *E. coli* (UPEC).

Illumina reads from a total of 169 isolates (accession numbers in Table 1) were mapped to the sequence type 131 (ST131) reference genome, SE15 (accession no. AP009378) (Toh *et al.*, 2010) using the mapping-based pipeline RedDog (available at https://github.com/katholt/RedDog). Briefly, RedDog uses Bowtie2 (Langmead *et al.*, 2009) to map short reads to the reference genome, and then uses SAMtools (Li *et al.*, 2009) to call SNPs (Phred score ≥30, read depth ≥5×). Consensus alleles at all SNP sites identified in the isolate collection were then extracted from each read set using SAMtools (Li *et al.*, 2009) (Phred score ≥20 and unambiguous homozygous base call; otherwise allele call set to unknown, ‘−’).

The resulting SNPs were filtered to include only those located within common genes (defined as genes with ≥95 % coverage in ≥95 % of the ST131 genomes analysed), yielding a total of 38 213 SNPs. The resulting SNP alignment was used as input to infer a maximumlikelihood (ML) tree using RAxML (yielding 100 % bootstrap support for all major nodes). The phylogeny was outgroup-rooted using the group comprising four closely related ST95 isolates. These had originally been identified as ST131 in PCR analysis for *rfb* and *pabB* genes, before MLST confirmed they were ST95 (Petty *et al.*, 2014).

### Analysis of GenomeTrakr data.

GenomeTrakr (NCBI BioProject: PRJNA183844) is a public repository of genome data from foodborne pathogens submitted by various laboratories including the US Food and Drug Administration and the Centers for Disease Control and Prevention. It includes raw Illumina reads and a kmer-based phylogeny of *E. coli* read sets, which is updated daily to incorporate newly submitted data. To demonstrate typing of foodborne isolates, we downloaded from GenomeTrakr a subset of the 300 most recently submitted read sets and the current kmer tree on 5 June 2015. A subtree representing relationships between the 300 downloaded isolates was extracted from the full kmer tree, by removing all other tips from the tree using R packages *ape* (Paradis *et al.*, 2004) and *Geiger* ( Harmon *et al.*, 2007).

For the serotype diversity analysis, an additional 1000 isolates from different sources were downloaded from GenomeTrackr. Briefly, the potential isolates were first filtered to those with SRA accession numbers and then by source, which were turkey (*n*=151), pig (*n*=110), human faecal (*n*=56), human urine (*n*=13), human blood (*n*=31), food (*n*=16), other miscellaneous animals (*n*=34), environment (*n*=262), chicken (*n*=225) and cattle (*n*=365). The last three source attributes were then randomly sampled for 196 isolates from chicken and cattle and 197 from the environment.

### Analysis of O- and H-antigen diversity in 1547 isolates.

Tbe program srst2 v0.1.7 was used to infer MLST and serotype for a total of 1547 isolates, using default settings. The isolate set comprised the 185 atypical EPEC (aEPEC) from the GEMS (Ingle *et al.*, 2016), 362 ETEC isolates (Mentzer *et al.*, 2014) and the 1000 isolates from GenomeTrakr. eBURST groups were identified from MLST data using Phyloviz (Francisco *et al.*, 2012) to cluster groups of STs separated by single locus variants (SLVs). For this analysis, novel SNP variants of known MLST alleles were ignored (i.e. treated as identical to the known allele, because we were interested in SLV-linked eBURST groups not individual novel STs). Entirely novel STs (novel combinations of known alleles, or combinations of novel alleles) were designated ‘Novel’. O- and H-types were assigned on the basis of confident calls against the EcOH database. Diversity analyses were performed using the *vegan* package for r (Oksanen *et al.*, 2015), excluding isolates for which there was no confident O-group call. Collector’s curves were generated using the function *rarecurve*. For isolates for which there were two H-types detected (*fliC* and non-*fliC*), the non-*fliC* allele was used to capture the diversity of H-types in the isolates. Variation within ST and eBURST groups by source, O-group, H-type and O:H serotype were explored through visualization of the data with heatmaps using the *pheatmap* package.

### Identification of novel O-antigen sequences in ETEC and GenomeTrakr isolates.

The initial run of the EcOH database yielded 239 isolates with no O-antigen confidently detected by srst2 in addition to seven aEPEC GEMS isolates with no O-antigen calls. For 72 isolates this was due to low read depth; these isolates also had poor *de novo* assemblies. For the remaining 167 isolates with no O genotype assigned, but with reasonable quality assemblies, we searched for novel O-antigen loci using the same approach as described above for GEMS isolates. The entire O-antigen region between *galF* and *gnd* was extracted from the *de novo* assembly. For 86 isolates, *galF* and *gnd* were on the same contig, yielding a complete O-antigen region sequence. These sequences were clustered using cd-hit (v4.6.4) (Fu *et al.*, 2012), with a cut-off of 0.90 (i.e. 90 % nucleotide similarity). These clusters were also compared with the prototype O-antigen sequences with the same method using cd-hitto confirm the novelty of the O-antigen locus structure. Three of these sequences were identified as allelic variants of prototypical O-antigens and were added to the EcOH database.

We also found 41 novel clusters, six of which were the novel GEMS O-antigens. The remaining sequences were then annotated with Prokka (v1.11) (Seemann, 2014) and putative *wzx* and *wzy* genes were analysed using tmhmm (Krogh *et al.*, 2001) to confirm that they contained transmembrane domain proteins. In representatives from three of the 35 novel clusters, no putative *wzx* or *wzy* genes could be confidently identified. These loci were added to the EcOH database and labelled as ‘wzx-novel1’ etc.

## Results and Discussion

Here we present the EcOH database for serotyping *E. coli* directly from short read data. We first sought to validate the EcOH database with publicly available *E. coli* genomes for which O- and H-types have been reported. We then used 197 recently sequenced EPEC isolates to validate serotyping against the EcOH direct from short read data using srst2, comparing the results with both serological phenotyping and blast-based analysis of genome assemblies. Having established the validity of our EcOH short read typing approach, we aimed to demonstrate its utility for typing endemic clones and foodborne outbreaks. To achieve this, we utilized two datasets. The first comprised 169 UPEC isolates that have been characterized previously for phylogenetic structure, serotype and antimicrobial resistance profiles, acting as a reference against which to evaluate our read-based typing approach. The second dataset comprised 300 isolates from the GenomeTrakr database of isolates sequenced for public health investigations. No MLST or serotype information is available for these isolates, although this dataset allowed us to demonstrate the power of our approach to characterize isolates of public health concern via rapid identification of both serotype and ST. Finally, this study sought to explore the serotype diversity in a large collection of *E. coli* genomic read sets. To this end we utilized three datasets, the 185 aEPEC isolates (that formed the bulk of the 197 EPEC isolates used for validation), 362 ETEC isolates and a total of 1000 isolates from GenomeTrakr, giving a total of 1547 *E. coli* genomes for analysis.

### Preliminary validation of the EcOH database

For 38 of the 40 genomes used for the preliminary validation of EcOH for O-groups, the expected O-group (or O-cluster based on near-identical gene clusters; Iguchi *et al.*, 2014) was detected using blast+ (Table S2). The two exceptions were *E. coli* isolates SE11 and SE15, which are reported in the literature as O152 and O150, respectively (Oshima *et al.*, 2008; Toh *et al.*, 2010). *In silico* analysis of these genomes identified *wzx* and *wzy* alleles for O16 and O173, respectively, and no blast+ hits to the alleles corresponding to serogroups O152 and O150, suggesting that the original serotypes were incorrect. Reported H-types were available for 20 of the reference genomes and we identified the expected H-alleles in all of these (Table S2), including both *fliC* H4 and *flnA* H17 in strain p12, which was consistent with a previous report of two flagellin loci in this isolate (Ratiner *et al.*, 2010).

### Comparison of serological phenotyping with *in silico* serotype prediction

We next compared *in silico* serotyping (i.e. O- and H-genotyping) of Illumina WGS data with serological phenotyping of 197 EPEC isolates. All these isolates were serotyped by a national reference laboratory in Melbourne, Australia, and yielded phenotypic identification of O-group for 144 isolates (73 %; total 44 O-groups). The remaining 53 isolates were assessed either as O-rough (*n*=9, the isolates auto-agglutinated or were hyper-mucoid) or as O-non-typeable (*n*=44, agglutination did not occur with any antiserum). H-types were phenotypically identified for 128 isolates (65 % of those tested; 18 different H-types). The remaining 69 isolates were identified as H- (*n*=67, indicating that they were non-motile) or H-rough (*n*=2, indicating non-specific agglutination with H-antisera).

As the 197 EPEC isolates had previously undergone WGS using the Illumina HiSeq platform (Ingle *et al.*, 2016), we compared two different strategies for *in silico* assignment of O- and H-types to these isolates using the EcOH database: (i) typing direct from reads using srst2, and (ii) *de novo* assembly (using Velvet Optimiser) followed by identification of alleles via blast+ (see Methods). The results are summarized in Table 2 with the full results reported in Tables S3–S5. Analysis via srst2 yielded matching (i.e. same O-group) confident genotype calls at two O-determining loci (either *wzx* and *wzy*, or *wzm* and *wzt*) for 167 isolates (85 %), and at one O-determining locus for a further 15 isolates. Thus, a total of 182 (93 %) isolates were genotyped using srst2, including 137/144 (95 %) of those that were serologically typeable and 45/53 (85 %) of those that were not (i.e. those which the reference laboratory identified as O-non-typeable or O-rough) (Table 2). In comparison, blast+ analysis of the Velvet Optimiser assemblies yielded full-length (>90 % coverage) hits to one or more O-gene locus for 180 (91 %) isolates, including 135/144 (94 %) of serologically typeable isolates and 45/53 (85 %) of non-typeable isolates (Table 2). Alternative assemblies generated using SPAdes yielded fewer hits, with only 91/144 (64 %) serologically typed isolates yielding full-length (>90 % coverage) blast+ hits to any O-gene in the EcOH database. These assemblies were not analysed further.

**Table 2.** Comparison of serotype calls in 197 EPEC isolates from serological phenotyping and *in silico* serotyping using the EcOH database with srst2 and blast+ Phenotype, srst2 and blast+ refer to the method used to determine O-group or H-type.The SRST2/BLAST+ column refers to isolates that received a confident genotype call using both the SRST2 and BLAST+ methods. The breakdown of *in silico* analysis into Phenotype and No phenotype refers to number of confident calls made for isolates with a serological phenotype and those without, respectively.

Antigen	Phenotype*	Genotype (EcOH)†					
O-group	Phenotype	**srst2**		**blast+**		**srst2/blast+**	
	144 (73 %)	182 (92 %)		180 (91 %)		179 (91 %)	
		Phenotype	No phenotype	Phenotype	No phenotype	Phenotype	No phenotype
		137	45	135	45	134	45
H-type	128 (65 %)	194 (95 %)		179 (91 %)		179 (91 %)	
		Phenotype	No phenotype	Phenotype	No phenotype	Phenotype	No phenotype
		127	67	120	59	120	59

*O-group or H-type determined by serological phenotyping in a reference laboratory.

**†**O-group or H-type determined by *in silico* analysis using the EcOH database.

Of the 15 isolates for which srst2 analysis did not yield a serotype call, seven had serological O-groups, but no high confidence calls for any *wzx*/*wzy* or *wzm*/*wzt* genes [serologically typed as O2 (*n*=3), O103 (*n*=1), O108 (*n*=1), O124 (*n*=1) or O153 (*n*=1)]. For six of these seven isolates, no O-antigen genes were detected in the assemblies either; for one isolate (serologically O103), srst2 yielded a low-confidence call of O111, while assembly analysis detected O103 *wzx* and *wzy* alleles (Table S3). The remaining eight isolates had no serotype detected via phenotypic or genotypic assays.

Of the 144 isolates that yielded a serological O-phenotype, genotyping based on confident srst2 calls at one or more O-gene locus matched the serologically identified O-group for 121 isolates (84 %), a different O-group for 16 isolates (11 %; 15/16 with matching calls for both O loci) and no result for seven isolates (5 %) (Table S3). Of the 15 isolates for which srst2 calls agreed at the two O loci but did not match the serological phenotype, assembly analysis identified the same O-group as srst2 in 14/15 cases, and the serological O-type in 1/15 cases. There was only one isolate for which assembly-based analysis identified the same O-group as phenotyping when srst2 had no result, and there were also two cases where srst2 analysis identified the serological O-group and blast+ did not.

Possible reasons for mismatches between O-antigen phenotype and genotype include multiple genetic variants manifesting in the same phenotype (e.g. O45, see Plainvert *et al.*, 2007) and/or atypical genetic variation such as multi-copy genes or novel genes. To explore these possibilities, we manually inspected the genome assemblies of isolates yielding conflicting genotype/phenotype calls and identified 12 novel O-antigen loci, which were added to the EcOH database with the suffix ‘var1, var2’, etc., to differentiate them from prototypical alleles (Fig. S1). For example, three isolates phenotyped as O116 or O33-related had detectable *wzx* O116 genes but no *wzy* genes. Interestingly, the *wzx* alleles were detected at high depth (~100×) and were highly divergent from the reference O116 *wzx* allele (~10 % nucleotide divergence, the maximum limit of detection we used for genotyping). We hypothesized that these isolates may carry *wzy* genes that are genetically distant from the prototypical alleles that were included in our database, but which nonetheless result in similar phenotypic agglutination patterns to isolates carrying more prototypical genes. Investigation of the corresponding genome assemblies revealed a novel O-antigen locus, including novel *wzx* and *wzy* variants that were 10 and 40 % divergent, respectively, from the prototypical O116 gene sequences (Fig. S1a). The novel *wzx* and *wzy* sequences were labelled O116var1 and added to the EcOH database to facilitate identification of this type in future. In the genomes of four isolates genotyped as O8 but phenotyped as O153 (Table S3), we confirmed the presence of O8 *wzx* and *wzy* alleles (Fig. S1b). The novel structures of these O-antigens compared with the prototype suggest convergent evolution to give the same phenotype, as shown previously for O45 (Plainvert *et al.*, 2007).

**Fig. 1.**
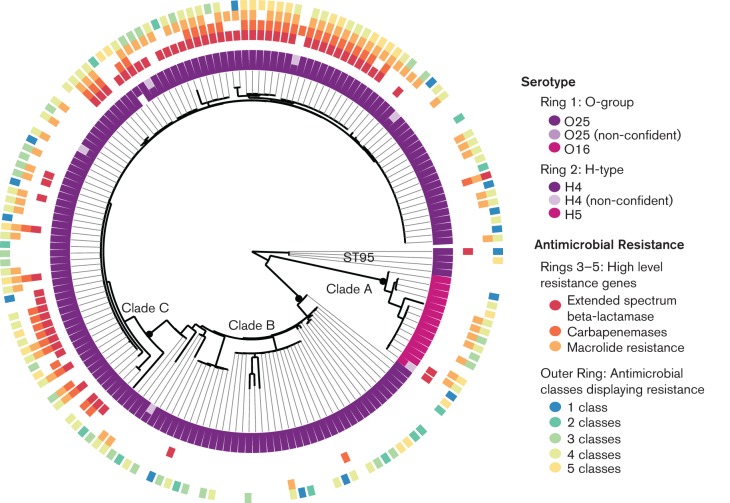
*In silico* prediction of serotype and antimicrobial resistance for UPEC ST131 isolates. Core genome SNP tree for 170 UPEC ST131 isolates, outgroup-rooted using ST95 isolates. Serotype and acquired antimicrobial resistance gene profiles detected using srst2 are indicated by rings surrounding the phylogeny. Low-confidence serotype calls, which are based on limited read depth, are shown in paler colours to indicate uncertainty.

H-typing using the EcOH database yielded similar results to O-ty ping. Analysis via srst2 returned 127 confident calls that matched the phenotype in 119 of 128 (93 %) of the serologically H-typed isolates, and gave confident genotype calls in 67 of 69 (97 %) non-motile (serologically H-) isolates (Fig. 1b, Tables S4 and S5). In contrast, assembly analysis identified the expected genes in only 112/128 (88 %) serologically H-typed isolates and 59/69 (86 %) non-motile isolates (Fig. 1d, Tables S4 and S5). The high rate of genotype calls amongst phenotypically H-non-motile isolates is probably due to a lack of flagellin expression during serotyping, which does not affect genotyping. Only two isolates had no flagellin genes detected from the sequence data. These were non-motile and may be the only isolates that genuinely lack the ability to express flagella.

The data above show that the use of srst2 and the EcOH database to type raw Illumina read sets can provide rapid *in silico* serotyping that outperforms assembly-dependent analysis (especially for H-typing) and is largely predictive of results obtained from serological typing while yielding fewer ‘untypeable’ results. In addition to the EcOH database, other databases, such as those used for MLST and antibiotic resistance gene profiles, can be interrogated using srst2 (Inouye *et al.*, 2014), with a single srst2 analysis returning MLST, serotype and antimicrobial resistance genotype results in approximately 5–10 min for paired Illumina data at mean read depth of 50–100× (Inouye *et al.*, 2014). We therefore sought to demonstrate the utility of this approach for the rapid characterization of *E. coli* genomes, including serotyping, MLST and antibiotic resistance gene profiling, in a variety of contexts.

### Applications of rapid *in silico* serotyping of *E. coli* to public health

To investigate the capacity of our approach to yield informative data in a public health setting, we analysed the whole genome read sets of 169 UPEC isolates previously reported as belonging to the epidemic UPEC clone ST131, which had also been characterized for serotype and antibiotic resistance profiles (Petty *et al.*, 2014; Price *et al.*, 2013) (Fig. 1). We confirmed most isolates were ST131. However, six isolates were single locus variants of ST131, including four belonging to ST95, consistent with the original report on these genomes (Petty *et al.*, 2014). *In silico* serotyping identified the majority of isolates (90 %) as O25:H4, which is the reported serotype of this epidemic clone (Nicolas-Chanoine *et al.*, 2008). However, we also identified isolates (8 %) as O16:H5. These clustered together tightly in the core genome phylogeny, indicating they represent a sub-clone of ST131 in which a change of serotype has occurred (Fig. 1). The O16:H5 sub-clone carried fewer resistance genes than other ST131 genomes and corresponds to ST131 Clade A, which has been identified as an ancestral sub-lineage of ST131 that is distinct from the sub-lineage which is now globally disseminated (Petty *et al.*, 2014). O-antigen variation within ST131 was detected in the original genome reports (Petty *et al.*, 2014; Price *et al.*, 2013), but was not explored in detail. Our data highlight the utility of *in silico* serotyping to illuminate ongoing microevolution in significant epidemic clones of *E. coli*, including change of serotype, which could confound serological identification of outbreak-related isolates.

**Fig. 2.**
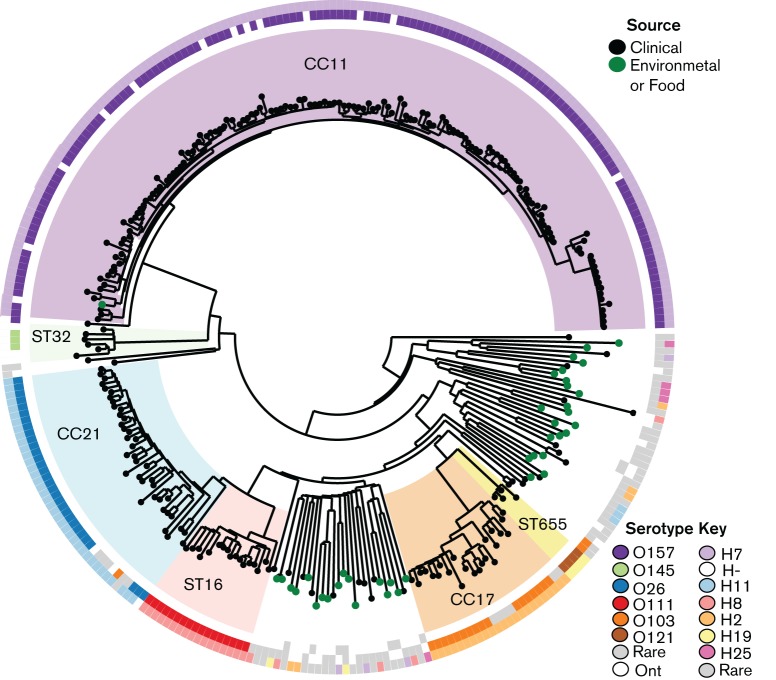
*In silico* prediction of serotype and ST from GenomeTrakr data. Shown is the kmer-based tree obtained from the GenomeTrakr project; common STs or CCs, identified via MLST analysis using srst2, are highlighted and labelled. Rings show predicted O-group (inner ring *wzm/wzt* genes and middle ring *wzx/wzy* genes) and H-type (outer ring).

Next we undertook *in silico* serotyping of 300 foodborne outbreak-associated *E. coli* genomes deposited by public health laboratories into the GenomeTrakr project (NCBI BioProject accession: PRJNA183844) to demonstrate the utility of our approach in characterizing isolates from short-read data alone without the need for additional processing. Fig. 2 shows our *in silico* serotyping results overlaid on the GenomeTrakr kmer-based tree. Environmental isolates displayed a diversity of ST, O- and H-types, whereas most clinical isolates belonged to one of six clonal lineages, each characterized by a specific serotype (Fig. 2). The predominant lineage amongst these recently deposited isolates was the well-characterized EHEC lineage ST11 O157:H7. Other lineages included ST16 O111:H8; ST655 O121:H19 and ST232 O145:H- (in which no serotype variation was detected), as well as clonal complexes (CCs) CC21 O26:H11 and CC17 O103:H2 (both of which displayed some serotype variation, Fig. 2). For 227 isolates (76 %), matching confident calls were obtained for both O-antigen genes, whilst 272 isolates (91 %) had a confident call for at least one allele. In most cases where a low-confidence O-antigen genotype call was made (due to low read depth), the call was for O157 alleles. The position of these genomes within the ST11 O157:H7 lineage of the kmer tree suggests that the low-confidence calls of these genomes are likely to be correct. Only seven isolates (2 %) yielded no genotype calls for the H-locus, indicating they are likely to be non-motile. These data demonstrate the utility of our method for *in silico* serotype prediction of *E. coli* sequenced for the investigation of foodborne outbreaks and also highlight the diversity in serotypes of isolates associated with such an outbreak.

### *E. coli* serotype diversity

To investigate the diversity of serotypes in the wider *E. coli* population, we analysed 1547 Illumina WGS read sets sourced from GenomeTrakr (including a wide range of human and animal isolates identified during the investigation of foodborne outbreaks) and two recent studies of global diversity amongst the human diarrhoeagenic pathotypes , ETEC and aEPEC (see Methods). Initial serotype prediction using srst2 and EcOH identified a total of 137 O-groups/O-group clusters, with 16 % of isolates showing no match to known O-groups. Detailed analysis of the *de novo* assemblies of these isolates revealed a further 32 novel O-antigen loci that had unique gene arrangements compared with known loci (Fig. S2, described in Methods). These were added to the EcOH database (in addition to the six novel loci identified amongst aEPEC described above). Hence, the EcOH database now contains 199 distinct O-groups/clusters, including 183 O-groups and 16 O-group clusters each containing two to five distinct O-groups (37 in total).

We ran srst2 on the 1547 read sets using the updated EcOH database for serotyping and simultaneous MLST profiling. This analysis identified 171 of 199 O-groups/O-group clusters (156 O-groups and 15 O-group clusters). In total, 1435 of 1547 isolates (93 %) had a confident call for one or more O-antigen typing genes, while 76 had low-confidence calls due to low read depth (mostly ST11 with low-confidence calls for O157:H7) and 36 isolates had no O-group assigned. Amongst the isolates with confident calls, we detected *wzm*/*wzt* genes and *wzx*/*wzy* genes in 46. For example, 21 isolates had O8 *wzm*/*wzt* genes in addition to O8, O104 or Gp6 *wzx*/*wzy* genes. A further four other isolates had *wzx* and *wzy* genes belonging to unrelated O-groups, for example O7 *wzx* and O15 *wzy*, suggesting possible partial recombination within the O-antigen region. We speculate that these isolates would probably be O-non-typeable by serology, due to cross-reactions with the antisera. However, our *in silico* approach was able to identify specific genetic variation underlying O-antigen diversity. Our analysis also identified 49 of 53 known H-types. Fourteen of the 1547 isolates harboured two H-types, including *flkA*, *fllA* and *flmA*. Only 26 isolates (1.7 %) yielded a non-confident H-type call. Fifteen of these showed low read-depth across the *fliC* locus, while the remaining 11 appeared to lack flagellin entirely.

**Fig. 3.**
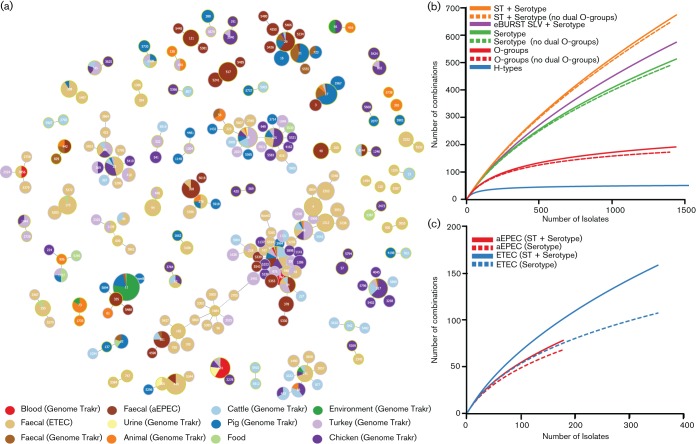
Population structure and collector’s curves showing genetic diversity among 1547 *E. coli* isolates. (a) eBURST analysis of MLST data for 1547 *E. coli* isolates identified 55 eBURST groups consisting of STs linked by SLVs (shown) and 178 unrelated STs (not shown). Circles indicate unique STs; SLVs (i.e. STs that share 6/7 alleles) are linked together to form eBURST groups. Circle sizes are proportionate to the number of isolates observed for each ST. The sources of isolates of each ST are shown in pie charts, using the colour code shown in the inset legend. (b) Collector’s curves illustrating the rise in the total number of distinct types observed as more *E. coli* isolates are included in the analysis. (c) Separate collector’s curves for two diarrhoeagenic *E. coli* pathotypes, aEPEC and ETEC.

MLST analysis linking SLVs clustered the 1547 isolates into 232 eBURST groups, including 55 clonal complexes comprising multiple STs (Fig. 3a). Only 23 eBURST groups (18 clonal complexes and five singleton STs) were common in our collection (represented by more than 10 isolates), with 70 % of isolates (*n*=1084) belonging to one of these common groups (Fig. 3a).

Next we calculated collector’s curves, based on the sample of 1547 genomes, to investigate how much additional serotype and lineage diversity one could expect from increased sampling of the *E. coli* population (Fig. 3b). The curves for O- and H-types each rapidly reached a plateau (~200 O-groups/clusters and ~50 H-types) (Fig. 3b). Although the isolates available for this analysis were subject to sampling bias, as most were associated with clinical or foodborne outbreaks, our data suggest that most of the O- and H-types of *E. coli* have been discovered and are included in the EcOH database. Deeper sampling of human commensal or environmental isolates would almost certainly yield many more strain types, although this is expected to result from novel combinations of O:H and strain background rather than a large number of novel O- or H-antigen loci.

Altogether we observed >500 O:H serotypes in our data sets, covering approximately 5 % of the possible O:H space of a hypothesized ~10 000 types. The collector’s curves for combined O:H serotypes were almost linear, indicating they are unlikely to be rapidly exhausted even with extensive increased sampling (Fig. 3b, c). Our sample did not have sufficient statistical power to test for dependence between O- and H-types. However, we did observe cases of the same O:H serotypes in distinct lineages of *E. coli*. This indicates convergent evolution resulting in the same serotypes occurring in highly divergent chromosomal backgrounds that are likely to differ in their gene content and pathogenic potential. This finding supports our contention that the value of *in silico* serotyping lies in combined analysis with core genome analysis such as ST or eBURST groups. For example, within the common ST10 group (*n*=64) there were 45 distinct serotypes, made up of 36 O-groups and 15 H-types. Isolates from different sources within lineages clustered within different serotypes (Fig. S3) indicating that serotype data allow the detection of micro-evolutionary events within lineages.

The collector’s curve of *E. coli* types (Fig. 3b) suggests the combination of surface antigens in different chromosomal backgrounds of *E. coli* is vast. If we define unique *E. coli* strain types as unique combinations of ST, O-group and H-type, we identified >700 such types amongst just 1547 isolates, and if we use eBURST group, O-group and H-type there were >600 strain types. This is reflected partly in the observed serotype variation within individual eBURST groups and STs, and suggests that serotype alone is not enough to identify and track *E. coli* lineages, but is valuable it detecting sub-lineages that may be associated with increased resistance (as in ST131) or pathogenicity.

Importantly, we identified extensive diversity of serotypes and STs amongst EPEC and ETEC isolates (Fig. 3c). Both of these pathotypes include a number of serotypes, and both are defined by accessory gene content, which has allowed them to emerge on multiple occasions through horizontal gene transfer (Ingle *et al.*, 2016; Mentzer *et al.*, 2014). Within aEPEC, the dominant serotypes include several that have not been previously reported in association with human disease, such as O88:H25 [found here in ST328 aEPEC, which has been isolated from cattle (Irino *et al.*, 2005)], and also occurred in three ETEC isolates of the same ST. However, we also identified two ST328 aEPEC isolates with different serotypes, indicating that O-antigen exchange is ongoing within this ST. While many eBURST groups included isolates from a range of different sources, including both animal and human pathogenic isolates (Fig. 3a), within these eBURST groups, isolates from different sources were often differentiated by their serotype. Indeed, none of the strain types observed amongst aEPEC or ETEC isolates from humans was also detected amongst isolates sourced from animals. This lends support to the idea that within a given *E. coli* lineage, serotype can be a useful marker for distinct sub-lineages that may differ in accessory gene content and thus pathogenic potential.

Exchange of surface antigens has been noted in many other species of bacteria. In host-associated bacteria it is likely that the duelling forces of (i) selection for retention of certain serotypes that offer colonization advantages in a particular niche and (ii) diversifying selection from host immunity or predation by phages or protozoa ultimately determine the level of recombination observed in different populations. In *Salmonella* (a sister genus of *E. coli*) there is strong conservation of surface antigens within many lineages, which is thought to be associated with long-term adaptation to different hosts. This results in long-term stable associations between serotype and lineage, to the extent that it has been proposed that MLST could be used instead of serotyping (Achtman, *et al.*, 2012). Here we observed that in *E. coli* the association between serotype and lineage is relatively less stable (Fig. S3). We hypothesize the O-group diversity detected within *E. coli* lineages is associated with diversifying selection on this important surface antigen, which is constitutively expressed and is a strong immunological target in all mammalian hosts (Donnenberg & Finlay, 2013). By contrast, we found that the H-antigen was more stably maintained within *E. coli* lineages, and the same is true in *Salmonella* (Achtman *et al.*, 2012). This is probably associated with weaker diversifying selection pressure compared with that acting on the O-antigen, as in both organisms flagella are not produced in the host environment where motility is not required, which naturally facilitates escape from flagellin-targeted immunological responses.

## Conclusion

This study has shown that *E. coli* O- and H-genotypes can be rapidly and accurately extracted direct from WGS reads. Our analysis allowed us to explore the diversity of serotypes within the species *E. coli* and identify 38 novel O-group loci. Serotyping in *E. coli* permits comparisons to be made with more than 70 years of historical data. In future epidemiological studies, however, the value of serotyping will most likely lie in the *in silico* characterization of microevolution within *E. coli* lineages, and the demonstration of sublineages therein. Our approach to *in silico* serotyping has advantages over both (i) serological phenotyping, which is resource-intensive in terms of time, labour and reagent costs, and fails to identify up to one-third of *E. coli* isolates, and (ii) *in silico* genotyping of short read WGS data using *de novo* assembly-based analysis, particularly for H-typing, as genome assembly is more computationally expensive, harder to standardize and more sensitive to variation in the underlying quality of sequence data. Our analyses also demonstrated heterogeneity within two pathotypes of importance to human health, and illustrate the limitations of using serotype to identify pathogenic lineages of *E. coli*. Importantly, srst2 uses unprocessed WGS reads and can be used to extract additional useful genotyping information including MLST, antimicrobial resistance and virulence genes. Overall our data demonstrate that EcOH and srst2 can be used to readily infer serotypes from large volumes of genome data currently being produced by GenomeTrakr and other public health networks as part of routine, high-throughput investigations of foodborne *E. coli* outbreaks. Our methods will become increasingly important in identifying the emergence of novel serotypes within outbreak clades that may signal a shift in the pathogen population during its dissemination (as we identified in ST131 UPEC) and may help to elucidate new lineages of *E. coli* associated with disease.
