# Corrigendum: *In silico* serotyping of *E. coli* from short read data identifies limited novel O-loci but extensive diversity of O:H serotype combinations within and between pathogenic lineages

**DOI:** 10.1099/mgen.0.000109

**Published:** 2017-08-01

**Authors:** Danielle J. Ingle, Mary Valcanis, Alex Kuzevski, Marija Tauschek​, Michael Inouye​, Tim Stinear, Myron M. Levine, Roy M. Robins-Browne, Kathryn E. Holt​

**Affiliations:** ^1^​ Department of Microbiology and Immunology, Peter Doherty Institute for Infection and Immunity, University of Melbourne, Parkville, Victoria 3010, Australia; ^2^​ Centre for Systems Genomics, University of Melbourne, Parkville, Victoria 3010, Australia; ^3^​ Department of Biochemistry and Molecular Biology, Bio21 Molecular Science and Biotechnology Institute, University of Melbourne, Parkville, Victoria 3010, Australia; ^4^​ Microbiological Diagnostic Unit Public Health Laboratory, Peter Doherty Institute for Infection and Immunity, University of Melbourne, Victoria 3010, Australia; ^5^​ School of BioSciences, University of Melbourne, Victoria 3010, Australia; ^6^​ Department of Medicine, University of Maryland School of Medicine, Baltimore, MD 21201, USA; ^7^​ Murdoch Childrens Research Institute, Royal Children’s Hospital, Victoria 3010, Australia

There was an error in Fig. 1 in the published article. In the key, under Antimicrobial Resistance, the orange colour indicates the presence of the OXA-1 class D β-lactamase (not Carbapenemases as originally labelled).

The authors apologise for any inconvenience caused.



